# TROY Modulates Cancer Stem-Like Cell Properties and Gefitinib Resistance Through EMT Signaling in Non–Small Cell Lung Cancer

**DOI:** 10.3389/fgene.2022.881875

**Published:** 2022-05-13

**Authors:** Linying Wu, Yuman Yu, Liming Xu, Xiaoling Wang, Jianying Zhou, Yuehong Wang

**Affiliations:** ^1^ Department of Respiratory Disease, The First Affiliated Hospital, College of Medicine, Zhejiang University, Hangzhou, China; ^2^ Department of Geriatrics, The First Affiliated Hospital, College of Medicine, Zhejiang University, Hangzhou, China; ^3^ Department of Pathology, The First Affiliated Hospital, College of Medicine, Zhejiang University, Hangzhou, China

**Keywords:** non–small cell lung cancer, drug resistance, TROY, epithelial–mesenchymal transition, RNA sequencing, cancer stem-like cells

## Abstract

Targeted therapy has made breakthrough progress in the treatment of advanced non–small cell lung cancer (NSCLC) in the last 20 years. Despite that, acquired resistance of epidermal growth factor receptor tyrosine kinase inhibitor (EGFR-TKI) is an urgent clinical problem. Our study established an acquired gefitinib-resistant cell line, which exhibited epithelial–mesenchymal transition (EMT) and stem cell–like properties. Transcriptional sequencing and bioinformatics analysis revealed that TROY was significantly increased in gefitinib-resistant cells. Gene set enrichment analysis (GSEA) showed EMT was the core enriched hallmark in the resistant cells. TROY siRNA interference could overcome the gefitinib resistance with the downregulated expression of EMT and CSC markers. In addition, immunohistochemistry indicated that TROY was overexpressed in tumor samples from patients who acquired resistance to first-generation EGFR-TKI without T790M mutation and the expression of TROY was associated with poor prognosis in LUAD. Here, we provided the potential role of TROY in the resistance of targeted therapy and a new strategy to overcome the acquired resistance to EGFR-TKI in NSCLC.

## Introduction

Lung cancer is the leading cause of cancer-related deaths with an estimated 1.8 million deaths in the world in 2020 (Sung et al., 2021). The most common type of lung cancer is lung adenocarcinoma (LUAD), which comprises about 50% of all lung cancers (Travis, 2011). Epithelial growth factor receptor (EGFR) mutation is an all-important oncogenic alteration, occurring in 16% of patients with advanced LUAD (Rosell et al., 2009). The activation of EGFR is closely associated with the development of non–small cell lung cancer (NSCLC). In recent years, EGFR-tyrosine kinase inhibitors (EGFR-TKIs), such as gefitinib, erlotinib, icotinib, afatinib, and osimertinib, have been used as first-line treatment for NSCLC patients with EGFR-activating mutations (Tian et al., 2022). Although they initially responded favorably, EGFR-mutated NSCLC patients eventually acquire resistance to EGFR-TKI in 8–16 months after first-line targeted drug therapy (Rotow and Bivona, 2017).

Several mechanisms have been reported to be relevant to acquired drug resistance of first-generation EGFR-TKI. T790M, a gatekeeper mutation, is recognized in approximately half of EGFR-TKI resistance cases (Kobayashi et al., 2005). The activation of bypass receptors, including mesenchymal–epithelial transition factor (MET) amplification, human epidermal growth factor receptor 2 (HER2) activation, and fibroblast growth factor receptor (FGFR) amplification; the transformation of phenotype, including epithelial–mesenchymal transition (EMT); and transformation to small cell lung cancer play an essential role in the tolerance of EGFR-TKI (Kim et al., 2015; Westover et al., 2018). Corresponding inhibitors can be used to deal with the secondary mutation or bypass pathway activation; however, there is no effective treatment for phenotypic transformation such as EMT. Research studies show that about 20% of patients develop EMT after first-generation EGFR-TKI acquired resistance, and the incidence of EMT in resistant patients without secondary T790M mutation is as high as 20%–60% (Uramoto et al., 2010; Sequist et al., 2011).

EMT is deemed relevant to the invasion and metastasis of tumors. Furthermore, recent studies showed that the process of EMT generates cells with properties of stem cells (Mani et al., 2008). Cancer stem-like cells (CSCs) display self-renewing and differentiation abilities; hence, CSCs are considered the cause of tumorigenesis, metastasis, and relapse (Huang et al., 2020). In addition, a lot of research studies have shown that conventional therapy often fails to eradicate cancer cells that have become CSCs *via* activation of the EMT program, thereby permitting CSC-mediated drug resistance and clinical relapse (Shibue and Weinberg, 2017).

Tumor necrosis factor receptor superfamily member 19 (TNFRSF19), also known as TROY, is a type I transmembrane glycoprotein. Of late, TROY was confirmed as a susceptibility gene for nasopharyngeal cancer and lung cancer (Bei et al., 2010; Hu et al., 2011), and TROY can bind to TGFβ receptor I and block TGFβ downstream signal Smad2/3, thereby promoting tumor growth in nasopharyngeal cancer (Deng et al., 2018). Furthermore, studies have shown that TROY is involved in cell differentiation and stemness. It regulates the differentiation from mesenchymal stem cells to osteoblast and adipocytes (Qiu et al., 2010). In the kidney, TROY + cells are 40 times more capable of forming organoids than TROY cells in the kidney (Schutgens et al., 2017), while TROY + gastric chief cells can be cultured to organoids in the gastric epithelium (Stange et al., 2013). Some recent reports have shown that TROY is overexpressed in malignant tumors such as melanoma and glioblastoma (Spanjaard et al., 2007; Paulino et al., 2010). Moreover, the expression of TROY promotes migration and therapy resistance in glioblastoma (Ding et al., 2020).

To explore the potential mechanism of gefitinib resistance in NSCLC, we analyzed the expression levels of mRNA in the gefitinib-resistant cell line, HCC827/GR, and its parental cell line, HCC827, by RNA-sequencing. Based on the bioinformatics results, we determined that EMT is an essential gefitinib-resistant mechanism, and TROY modulates CSC properties and gefitinib resistance through EMT in T790M negative NSCLC.

## Materials and Methods

### Cell Lines and Culture Conditions

HCC827 cells were obtained from American Type Culture Collection and were grown in the recommended medium at 37°C with 5% CO_2_ in a humidified incubator. The gefitinib-resistant HCC827/GR cells were established by gradient dose treatment for parental cells for more than 6 months.

### RNA-Sequencing

After extraction, quantification, and purification, 10 μg of total RNA, in which ribosomal RNA was depleted, was fragmented into small pieces using divalent cations under elevated temperature. Following this, RNA fragments were reverse-transcribed to establish the final cDNA library according to the manufacturer’s instructions for the mRNA-seq sample preparation kit (Illumina). Then, paired-end sequencing was performed following the vendor’s recommended protocol on an Illumina Hiseq 4000 (LC-bio).

First, the reads that contained adapter contamination, low-quality bases, and undetermined bases were removed by Cutadapt, and the sequence quality was validated using FastQC (http://www.bioinformatics.babraham.ac.uk/projects/fastqc/). Then, Bowtie2 (Langmead and Salzberg, 2012) and Tophat2 (Kim et al., 2013) were used to map reads to the human genome, and then they were assembled by StringTie (Kim and Salzberg, 2011). All transcriptomes were combined to rebuild a comprehensive transcriptome using Perl scripts. Finally, StringTie and Ballgown (Zhang et al., 2016) were applied to estimate the expression levels of all transcripts.

The expression values of mRNAs were calculated as fragments per kilobase per million (FPKM) using StringTie (Pertea et al., 2015). The differentially expressed mRNAs (DE mRNAs) were chosen with a fold change (FC)≥2 or ≤0.5 and *p* ≤ 0.05 using the R package-Ballgown (Frazee et al., 2015).

### Enrichment Analysis

To understand the biological functions of DE mRNAs, Gene Ontology (GO) and Kyoto Encyclopedia of Genes and Genomes (KEGG) pathway enrichment analyses were performed by using the “clusterProfiler” package in R software. GO analysis includes cellular components, molecular functions, and biological processes, which explains the biological function of genes from different perspectives. The KEGG pathway enrichment analysis was used for analyzing the degree of enrichment of DE mRNAs in pathway terms.

Gene Set Enrichment Analysis (GSEA) was conducted to explore the characteristics of gene hallmarks in gefitinib-sensitive and gefitinib-resistant cells by using OmicStudio tools at https://www.omicstudio.cn/tool. In addition, h.all.v7.2.symbols.gmt was used as the reference gene set. Gene set was considered to be significantly enriched when a normal *p* value was <0.05 and a false discovery rate (FDR) was <0.25.

### RNA Interference

HCC827/GR cells were seeded in 6-well plates at a density of 1 × 10^5^ cells/well and replaced with a serum-free medium when the confluence reached about 60%–80%. 10 μl TROY siRNA or negative control siRNA (GenePharma) and 10 μl Lipofectamine RNAiMAX (ThermoFisher Scientific) were diluted in 150 μl Opti-MEM (ThermoFisher Scientific). Then, the diluted siRNA was mixed with the diluted RNAiMAX reagent and incubated for 5 minutes at room temperature. The siRNA–lipid complex was added to HCC827/GR cells and incubated for at least 24 h before the subsequent experiments.

### Cell Viability Assay

Parental and resistant HCC827 cells plated in triplicate were incubated with gefitinib for 72 h at distinct concentrations. Cell viability was measured with the cell counting kit-8 (CCK-8) assay (Dojindo Molecular Technologies) following the manufacturer’s protocol. At least three replicates were tested for each cell line on different days. The IC_50_ values were calculated by CalcuSyn software.

### Western Blot

The cells were lysed in a lysis buffer containing protease and phosphatase inhibitors, and cell lysate protein concentrations were determined using the BCA Protein Assay Kit (Thermo Fisher Scientific). Then, equivalent quantities (30 μg) of protein were separated with 8%–15% sodium dodecylsulfate-polyvinylidene gel electrophoresis and transferred to PVDF membranes (Merck Millipore). Then, they were probed with antibodies against TROY or Ki67 from Abcam and E-cadherin, N-cadherin, vimentin, ZEB1, ALDH1, ZO-1, or GAPDH from Cell Signaling Technology. After being incubated with secondary antibodies, the proteins were detected using an ECL kit (Beyotime Biotechnology).

### Sphere Formation Assay

Single-cell suspensions were plated at a density of 3000 cells in 6-well ultra-low-attachment plates (Corning) and cultured with DMEM/F12 (Gibco) supplemented with 20 ng/ml bFGF (PeproTech), 20 ng/ml EGF (PeproTech), 5 ng/ml heparin sodium (PeproTech), and 10% serum replacement (ThermoFisher Scientific) at 37°C in a humidified 5% CO_2_ incubator for 7–14 days. The number of spheres in microscopic field was counted using Image-Pro Plus 6.0 software. The median number from three replicates was used for statistical analysis.

### Immunohistochemistry Staining and Evaluation

The expression of TROY, E-cadherin, and vimentin in lung cancer tissues was detected by IHC staining from nine NSCLC patients who obtained resistance after first-generation TKI therapy. The samples were used with the approval of the Ethics Committee of the First Affiliated Hospital of Zhejiang University. After prepared into 4-μm-thick-sections, the tissue samples were deparaffinized, rehydrated, and unmasked antigens. The samples were incubated with primary antibodies (TROY 1:200 dilution, Abcam; E-cadherin and vimentin 1:500 dilution, Cell Signal Technology) at 37°C for 1 h. After washing in PBS, the sections were stained with DAB and counterstained with hematoxylin. Then, the IHC images were captured and scored by the distribution and immunoreactivity. The final results were determined by two observers independently. The percentage of positive cells was recorded as 0 if no cell was stained, 1 for less than 10% of stained cells, 2 for 10%–50% of stained cells, and 3 for more than 50% of stained cells. The staining intensity was recorded as 0 if no staining was observed, 1 for weak staining, 2 for moderate staining, and 3 for strong staining. Then, these values were multiplied together to provide a staining index for each case. Using this method of assessment, we evaluated the IHC score of TROY, vimentin, and E-cadherin in NSCLC by staining index (scored as 0, 1, 2, 3, 4, 6, or 9).

### Statistical Analysis

The data *in vitro* were presented with mean values ±SD from three or more independent repetitions. Two-tailed unpaired Student’s t-test was conducted to determine *p* value, and *p* < 0.05 was defined as statistical significant. Clinical data were analyzed using the chi-squared test or the two-sided Student’s t-test by SPSS Statistics 22 software. The Kaplan–Meier method was applied for survival analysis, and the log-rank test was performed to detect the significance of the difference between survival curves. Multivariate analysis was used to assess the prognostic value.

## Results

### Bioinformatics Analyses

**FIGURE 1:**
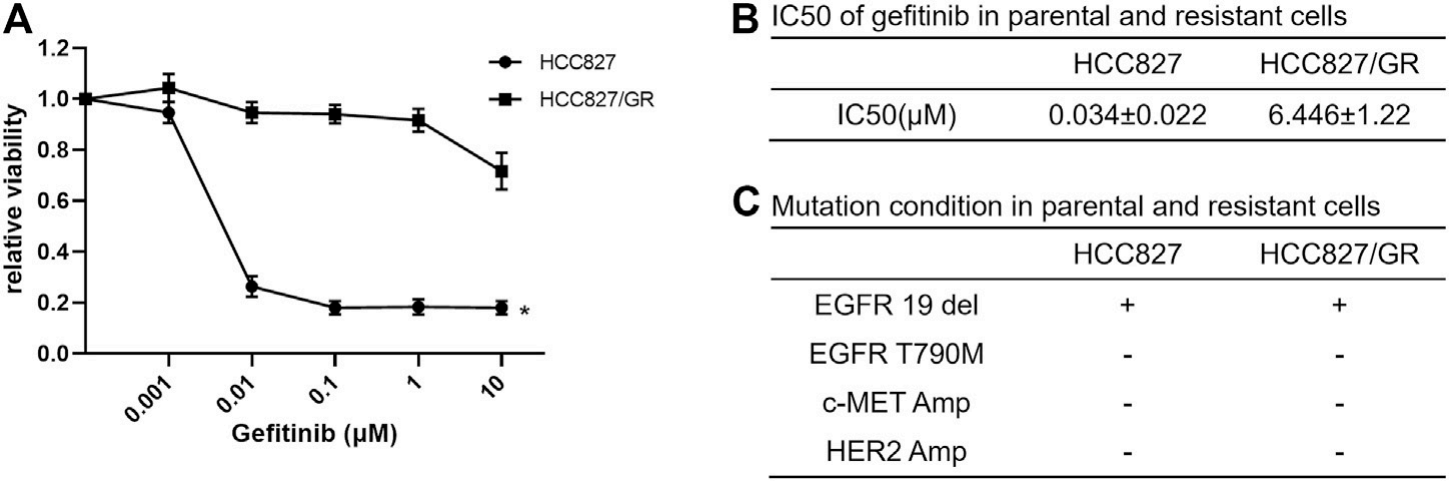
Establishment of gefitinib-resistant NSCLC cells. **(A)** Dose-response curves showed the effect of gefitinib on the viability of HCC827 and HCC827/GR cells. The cells were plated in triplicate and treated with the indicated doses of gefitinib for 72 h; then cell viability was measured by CCK-8 assay. Data are presented as mean values and standard deviations (SD) *p < 0.05 compared with the control. **(B)** IC_50_ of gefitinib in sensitive and resistant cell lines was calculated using CalcuSyn software. **(C)** Mutation state in gefitinib-sensitive and -resistant HCC827 cells.

To investigate the reasons for acquired resistance to EGFR-TKI, we established gefitinib-resistant NSCLC cell (HCC827/GR) by increasing the concentration of gefitinib in the culture medium gradually. The sensitivity of parental and resistant cells for gefitinib was detected by CCK-8 assay, and the IC_50_ of resistant cells was steeply increased compared with that of parental cells (Figures 1A,B). In addition, T790M mutation in exon 20 of EGFR, c-MET amplification, and HER2 amplification had not been detected in HCC827/GR cells (Figure 1C).

**FIGURE 2:**
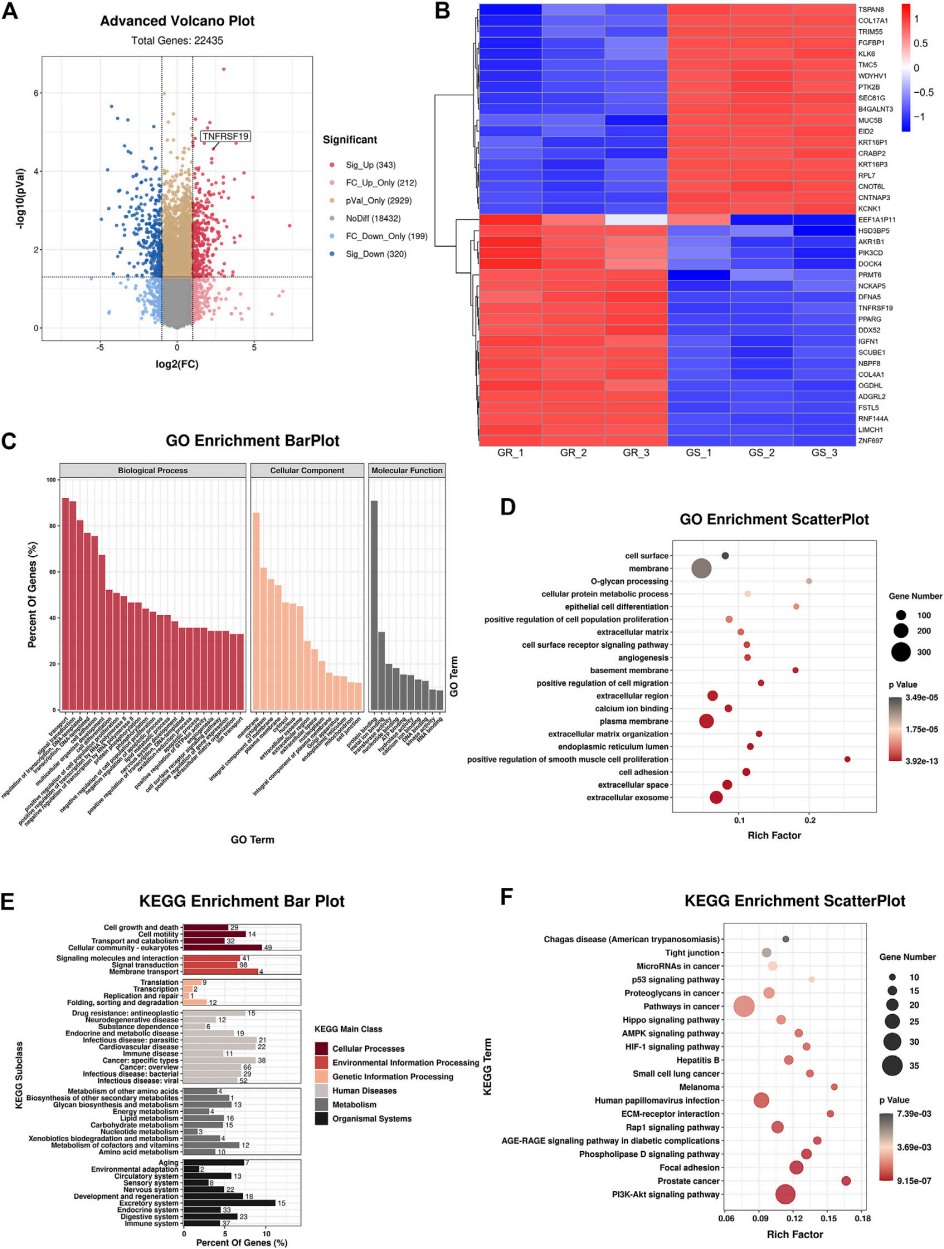
Bioinformatics analyses of HCC827 and HCC827/GR cells. **(A)** Advanced volcano plot showed the dysregulated mRNAs. **(B)** Heat map analysis indicated the top 40 dysregulated mRNAs. **(C)** GO terms of biological process, cellular component, and molecular function. **(D)** GO enrichment analysis. **(E,F)** KEGG analysis revealed significantly enriched pathways.

Then, RNA-sequencing was conducted to explore the expression level of mRNA in gefitinib-resistant cell and their parent cell line. The differentially expressed mRNAs were selected with a fold change ≥2.0 or≤0.5 and with statistical significance (*p* value < 0.05). The volcano plot showed that there were 343 upregulated mRNAs and 320 downregulated mRNAs (Figure 2A), and TNFRSF19 (TROY) mRNA was strikingly overexpressed in gefitinib-resistant cells. The heat map analysis displayed the expression of the top 40 dysregulated mRNAs (Figure 2B), and the mRNAs were listed in order of ascending *p* value (Table 1).

**TABLE 1:** Top 40 differently expressed mRNAs in gefitinib-resistant cell compared with parental cells.

Gene name	log2 (fc)	*p* values	q values	regulation
ADGRL2	3.02	0.0000002	0.01	up
SEC61G	−4.27	0.0000022	0.02	down
KRT16P1	−3.87	0.0000045	0.02	down
FSTL5	1.18	0.0000047	0.02	Up
FGFBP1	−3.21	0.0000049	0.02	down
LIMCH1	2.12	0.0000057	0.02	Up
CNTNAP3	−1.52	0.0000072	0.02	down
SCUBE1	1.97	0.0000078	0.02	Up
RNF144A	1.16	0.0000146	0.02	Up
ZNF697	2.38	0.0000158	0.02	Up
PPARG	1.01	0.0000181	0.03	Up
PRMT6	3.82	0.0000190	0.03	Up
DDX52	1.76	0.0000191	0.03	Up
PIK3CD	1.03	0.0000223	0.03	Up
COL17A1	−3.43	0.0000225	0.03	down
MUC5B	−1.68	0.0000262	0.03	down
TNFRSF19	2.35	0.0000271	0.03	Up
TMC5	−3.18	0.0000311	0.03	down
TRIM55	−3.02	0.0000340	0.03	down
WDYHV1	−1.43	0.0000461	0.04	down
HSD3BP5	2.24	0.0000479	0.04	Up
B4GALNT3	−2.23	0.0000563	0.04	down
AKR1B1	2.24	0.0000606	0.04	Up
KRT16P3	−2.17	0.0000782	0.05	down
PTK2B	−1.35	0.0000802	0.05	down
DFNA5	1.40	0.0000820	0.05	Up
RPL7	−2.00	0.0000843	0.05	down
DOCK4	1.22	0.0000850	0.05	Up
KCNK1	−1.43	0.0000884	0.05	down
KLK6	−4.52	0.0000920	0.05	down
NCKAP5	1.37	0.0000929	0.05	Up
IGFN1	4.33	0.0001098	0.05	Up
NBPF8	1.14	0.0001159	0.05	Up
OGDHL	1.65	0.0001189	0.05	Up
EID2	−2.64	0.0001203	0.05	down
TSPAN8	−2.68	0.0001288	0.05	down
CRABP2	−2.86	0.0001361	0.05	down
COL4A1	2.99	0.0001412	0.05	Up
EEF1A1P11	1.14	0.0001467	0.06	Up
CNOT6L	−1.16	0.0001592	0.06	Down

In addition, GO and KEGG analyses were carried out to examine the underlying functions of DE mRNAs. The GO analysis exhibited that dysregulation of mRNAs was associated with the plasma membrane, extracellular space, extracellular region, extracellular exosome, cell adhesion, and extracellular matrix organization (Figures 2C,D). Correspondingly, KEGG analysis demonstrated that the most significantly associated pathways were PI3K-Akt signaling pathway, focal adhesion, phospholipase D signaling pathway, and ECM-receptor interaction, among others (Figures 2E,F).

**FIGURE 3:**
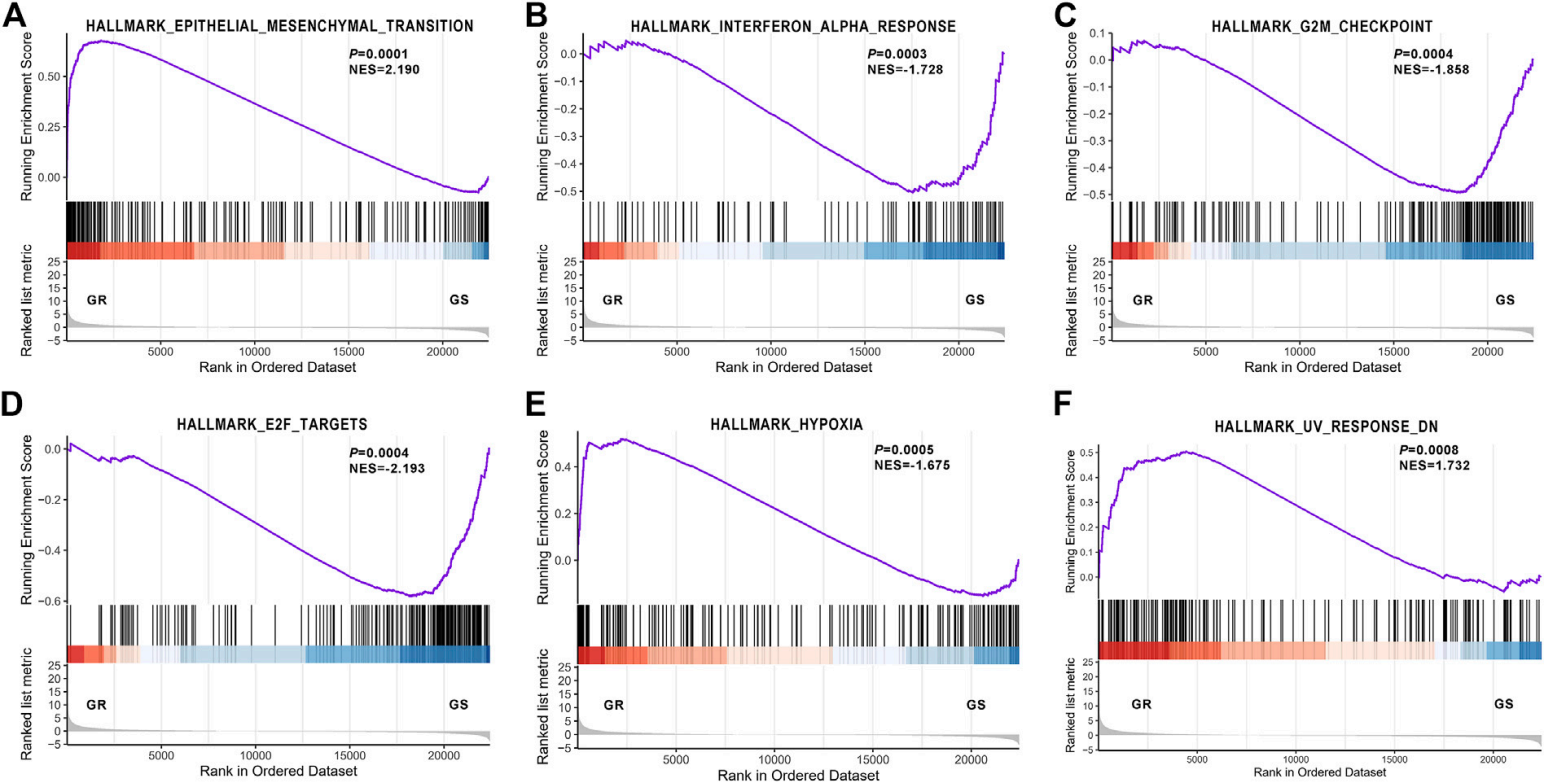
Gene Set Enrichment Analysis revealed the most significantly associated pathway. **(A)** Epithelial–mesenchymal transition; **(B)** Interferon alpha response; **(C)** G2M checkpoint; **(D)** E2F targets; **(E)** hypoxia; **(F)** UV response.

In addition, GSEA unveiled that EMT, interferon alpha response, G2M checkpoint, E2F targets, hypoxia, and UV response were the top six relevant pathways (Figure 3), and EMT was the overriding cause of gefitinib resistance.

### TROY Expression Affected Gefitinib Resistance in NSCLC Cells

**FIGURE 4:**
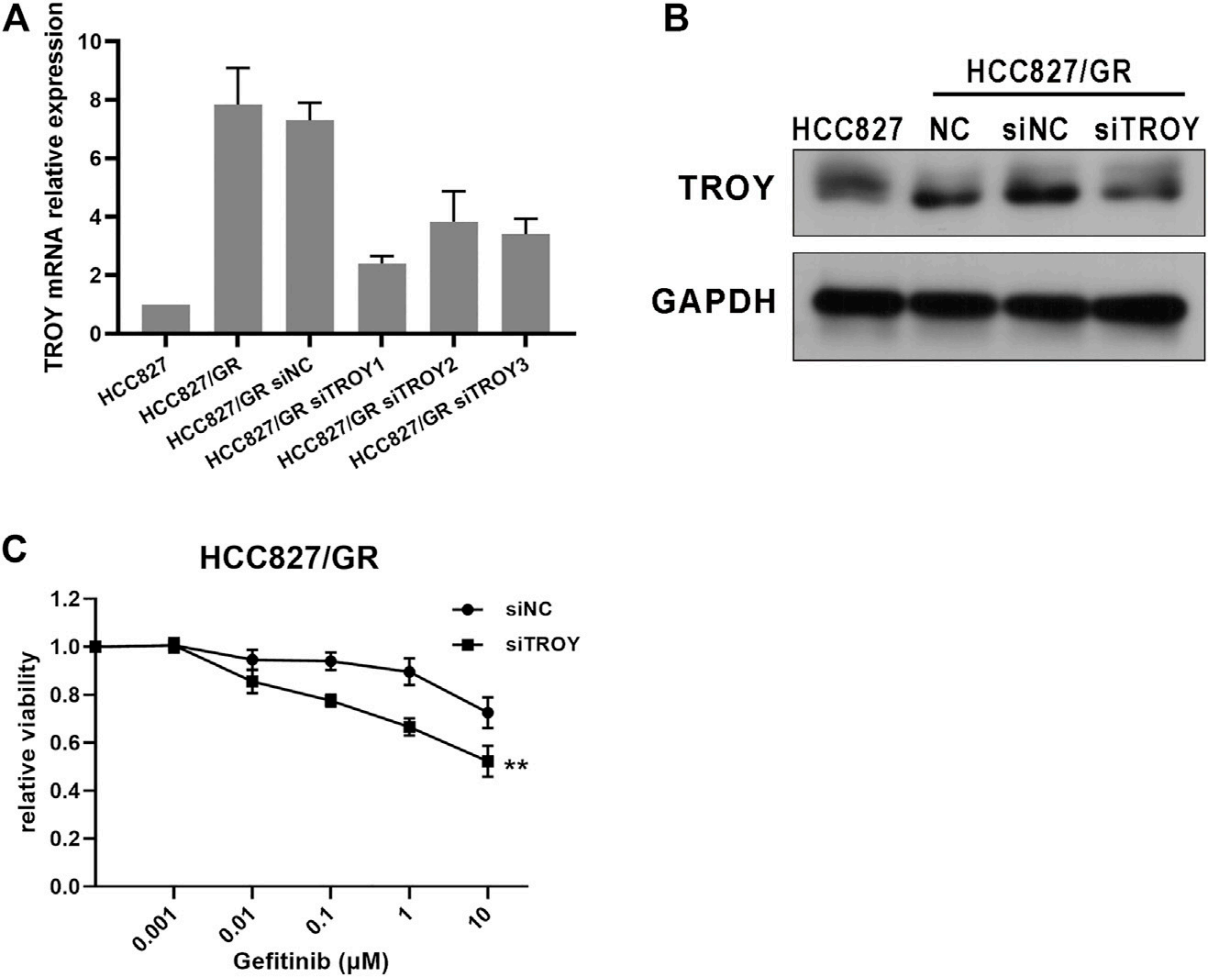
Knocked-down TROY enhanced the sensitivity of gefitinib-resistant cells. The mRNA and protein expression of TROY were detected by RT-PCR **(A)** and Western blot **(B)** in parental and resistant cells after 48 h incubation of the siRNA–lipid complex. **(C)** HCC827/GR cells transfected with siNC or siTROY for 24 h were treated with gefitinib at different concentrations, and then cell viability was detected using CCK-8 assay. **p < 0.01.

To investigate the role of TROY in gefitinib-resistant NSCLC cells, we first examined the expression of TROY in gefitinib-sensitive and -resistant NSCLC cell lines. Unsurprisingly, the mRNA and protein expression were both increased in gefitinib-resistant cells when compared to parental cells (Figures 4A,B). Then, we knockdown TROY by siRNA, and the results showed that TROY siRNA can effectively reduce TROY protein expression (Figure 4B), and cell growth was significantly reduced in TROY knockdown HCC827/GR cells when treated with gefitinib (Figure 4C), indicating that TROY is one of the reasons for gefitinib acquired resistance in NSCLC.

### TROY Was Associated With the EMT Process in Gefitinib-Resistant NSCLC Cells

**FIGURE 5:**
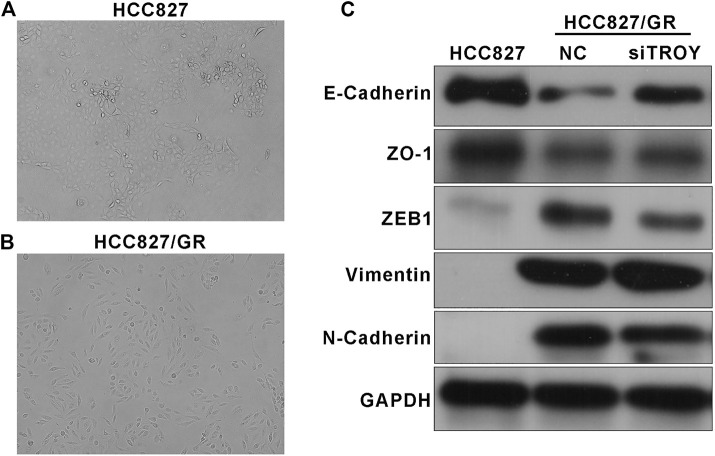
TROY was associated with the EMT process in gefitinib-resistant NSCLC cells. Representative photographs of HCC827 **(A)** and HCC827/GR **(B)** cell morphologies (magnification: ×100). **(C)** Western blot results showed an obvious drop in epithelial-related proteins E-cadherin and ZO-1 and a raise in mesenchymal-related proteins N-cadherin, vimentin, and ZEB1 in HCC827/GR cells. TROY siRNA transfection in HCC827/GR cells slightly reversed this process.

Phenotypic change was observed in acquiring resistant cells. Microscopically, HCC827/GR cells exhibited spindle cell–like morphology which differs from parental HCC827 (Figure 5A). It was foreseeable that HCC827/GR cells displayed EMT features with the decreased expression of E-cadherin and ZO-1 and the increased expression of N-cadherin and vimentin. In addition, ZEB1, an EMT transcriptional regulator, was upregulated too (Figure 5B). Since EMT has been identified as an indispensable reason for drug resistance in tumors, we detected the EMT features in TROY knockdown HCC827/GR cells. Interestingly, EMT‐related proteins N-cadherin, vimentin, and ZEB1 were significantly reduced after transfecting with TROY siRNA, and the expression of E‐cadherin and ZO-1 was elevated (Figure 5B). It suggested that TROY may play a crucial role in the EMT process in acquired resistance of EGFR-TKI.

### TROY Was Related to CSCs Phenotype in Gefitinib-Resistant NSCLC Cells

**FIGURE 6:**
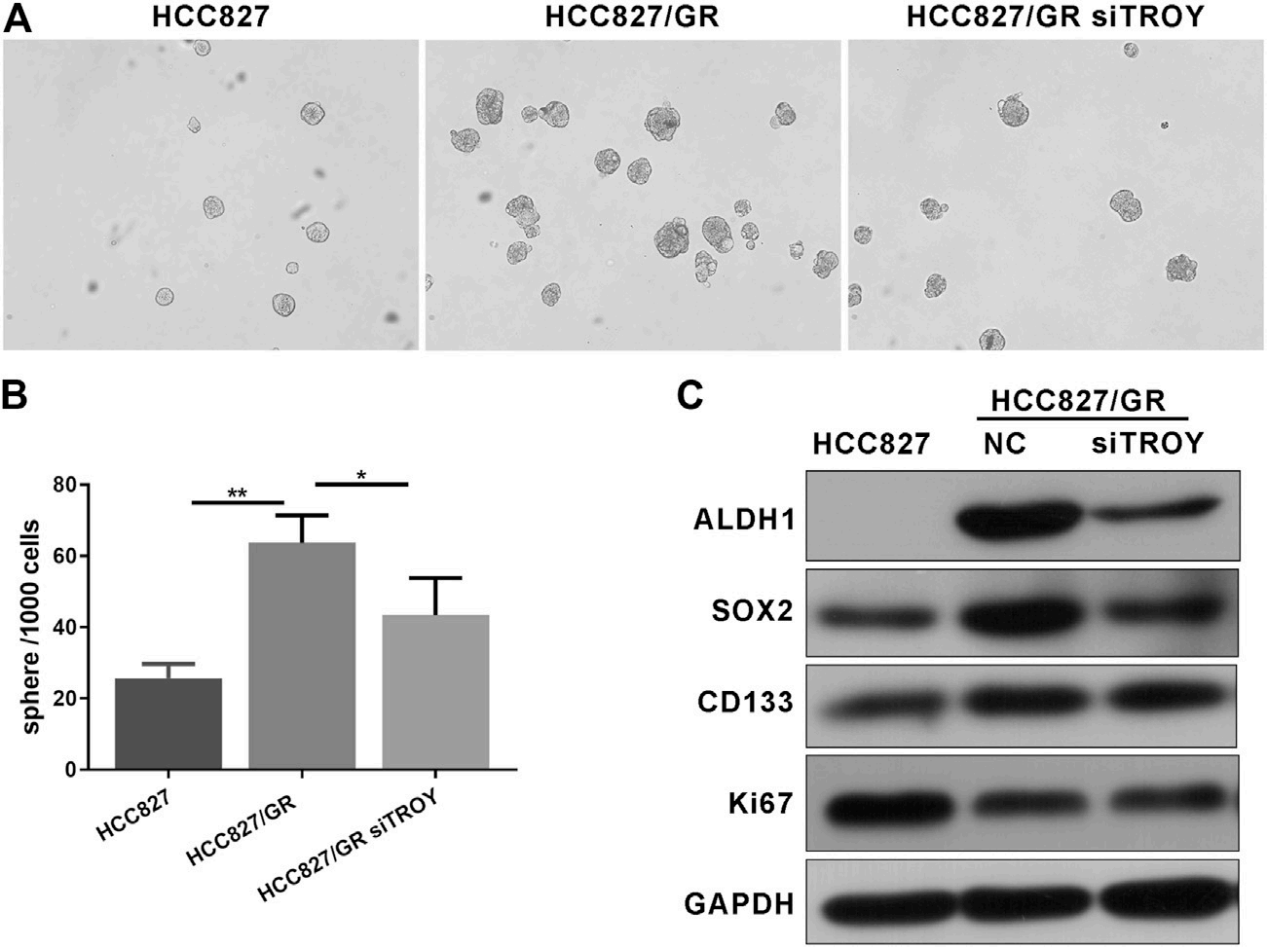
TROY was related to CSC phenotype in gefitinib-resistant NSCLC cells. Representative microphotographs of sphere formation **(A)** and the quantitative analysis **(B)** (Magnification: ×100). **(C)** Western blot analysis showed that ALDH1, SOX2, and CD133 proteins expression was raised in HCC827/GR cells when compared to HCC827 cells and was decreased after knock down TROY (mean ± SD, *p < 0.05, **p < 0.01).

Increasing evidence manifests that EMT is related to the obtainment of CSC features, in consequence, conferring resistance to cancer therapy. Sphere-formation assay is commonly used to detect cancer stem cell characteristics; thus, we compared the abilities of sphere formation between parental and resistant HCC827 cells. The results showed that gefitinib-resistant HCC827/GR cells displayed higher abilities of tumor sphere formation, while knockdown of TROY weakened the sphere-formation abilities in HCC827/GR cells (Figures 6A,B). Consistently, pluripotency markers, such as ALDH1, CD133, and SOX2, were elevated to varying degrees in HCC827/GR cells. In addition, Ki67, a marker indicating the degree of cell proliferation, was observed to be downregulated in HCC827/GR cells. Likewise, downregulated TROY gave rise to downregulated expression of stemness markers in gefitinib-resistant cells (Figure 6C). These data indicate that TROY may play a key role in the CSC regulation through EMT in NSCLC.

### Elevated TROY and EMT Features in Gefitinib-Resistant NSCLC Patients

**FIGURE 7:**
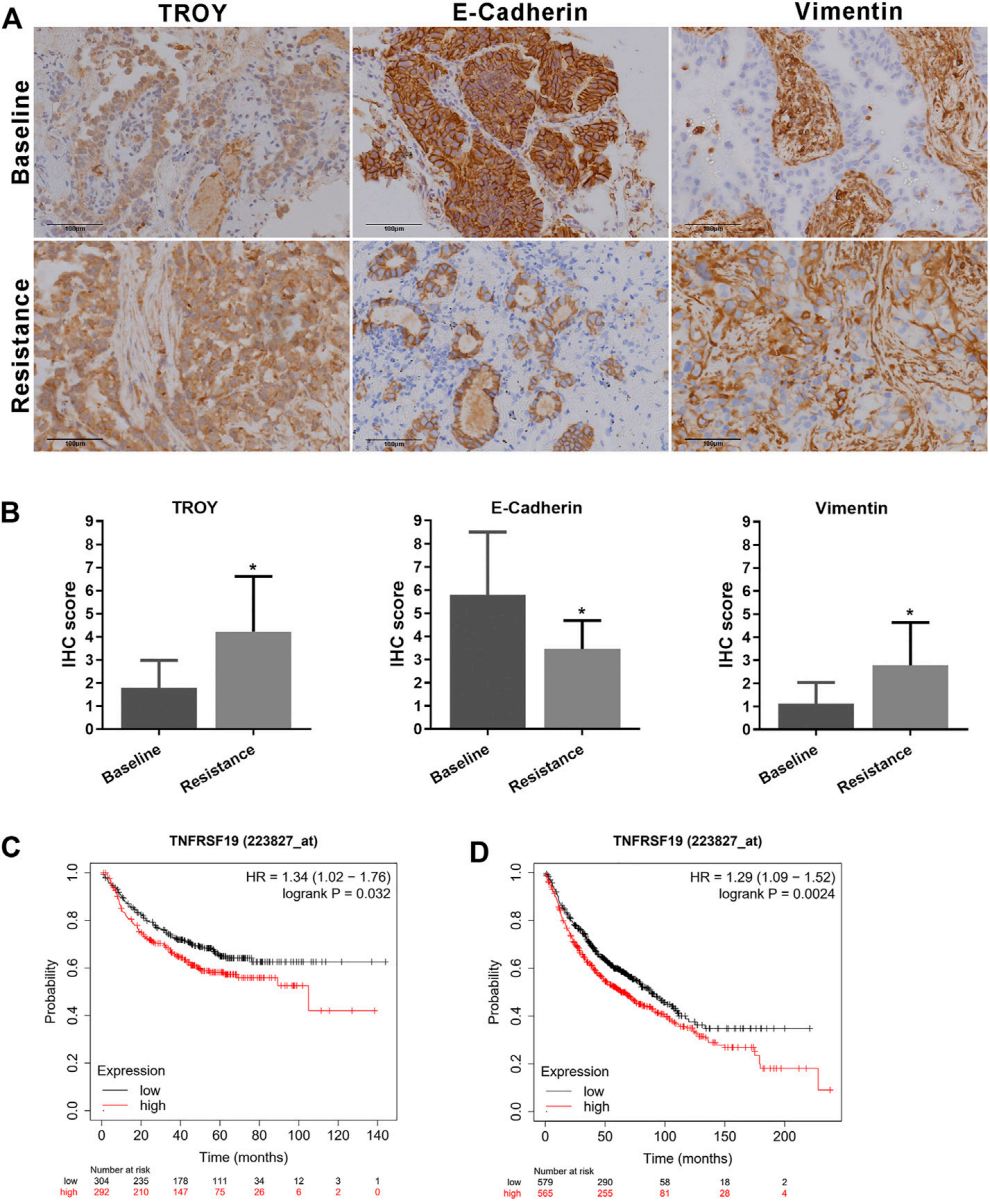
Elevated TROY and EMT features in gefitinib-resistant NSCLC patients. **(A)** Protein expression of TROY, E-cadherin, and vimentin in tumor samples from patient-resistant to first-generation EGFR-TKI and their baseline samples. Magnification: ×200. **(B)** IHC scores were used to evaluate TROY, E-cadherin, and vimentin IHC staining and were calculated for every patient. Data are presented as the mean ± SD, *p < 0.05 compared with baseline scores. First progression **(C)** and overall survival analysis **(D)** of LUAD patients with different expressions of TROY were conducted by the online tool Kaplan-Meier Plotter.

The expression of TROY, E-cadherin, and vimentin in tumor samples obtained from nine NSCLC patients who were resistant to the first-generation EGFR-TKI and without T790M mutation were determined by immunohistochemistry staining (Figures 7A,B). The expression of E-cadherin was found in all tumor tissues before treatment and decreased in patients after EGFR-TKI resistance with the expression of vimentin elevated. Concurrently, the expression of TROY was found to increase in patients after drug resistance (Table 2).

**TABLE 2:** Expression of TROY, E-cadherin, and vimentin in NSCLC patients before and after EGFR TKI resistance.

Patient ID	Age	Sex	EGFR mutation	TROY IHC score		E-cadherin IHC score		Vimentin IHC score		PFS (month)
				Baseline	Resistance	Baseline	Resistance	Baseline	Resistance	
1	60	M	Del 19	2	6	6	3	1	4	8
2	55	F	L858R	1	4	2	2	0	0	13
3	49	M	Del 19	0	2	4	4	0	1	15.5
4	44	M	Del 19	2	2	4	3	2	3	12
5	69	M	Del 19	3	4	9	3	1	6	7
6	57	M	Del 19	0	2	6	4	1	1	19
7	48	F	L858R	3	9	9	6	1	3	2.5
8	65	M	L858R	2	3	3	2	3	3	9
9	67	F	L858R	3	6	9	4	1	4	8

Survival analysis of TROY was conducted by the online tool Kaplan–Meier Plotter (https://kmplot.com/) (Győrffy et al., 2013). The first progression (FP) was 32.9 and 20.1 months in TROY-low expression and TROY-high expression LUAD patients, respectively. Meanwhile, overall survival (OS) was 88.7 and 63 months in TROY-low expression and TROY-high expression patients, respectively. In addition, multivariate analysis confirmed that TROY expression (HR = 1.29, 95% CI 1.09–1.52) was an independent prognostic factor in LUAD (Figures 7C,D). Either FP or OS was closely related with the expression of TROY in LUAD patients, which means TROY could be used as a prognosis biomarker for NSCLC.

## Discussion

NSCLC is a malignant tumor with high morbidity and mortality. Since the EGFR gene was initially identified as the driver of the development of NSCLC, many drugs that targeted EGFR have been designed. The development and wide application of EGFR-TKI molecular targeting drugs have greatly improved the PFS and survival quality of patients with EGFR mutation in lung cancer (Hsu et al., 2018). Nevertheless, therapeutics with EGFR-TKI drives the emergence of acquired resistance which poses a considerable hurdle with respect to EGFR targeted therapy. Gefitinib is a reversible first-generation EGFR-TKI that acts as an ATP competitive inhibitor and binds to the tyrosine kinase pocket of EGFR, thereby inhibiting EGFR phosphorylation and its downstream signaling pathway, discouraging the growth and inducing apoptosis of tumor cells (Morin, 2000). EGFR T790M mutation is a leading cause of acquired resistance to gefitinib, and the third-generation TKIs were recognized to be effective in this drug resistance mutation (Mayor, 2017). Drugs targeting other secondary mutations such as c-MET amplification and HER2 amplification are already available. Conversely, there is still no good solution for patients who takes first-generation TKIs and consequently get disease progression without EGFR T790M mutation. In this study, we established gefitinib, the first-generation EGFR-TKI, resistant cell line, which with no targetable mutations to explore the other mechanisms of EGFR-TKI resistance.

With the invention of RNA-sequencing, diagnosis and identification of cancer entered a new phase that enabled more accurate analysis and treatment of the tumors. RNA-sequencing could be used to detect chemotherapy-resistant cancer cells to identify potential mechanisms of drug insensitivity (Kong et al., 2020), predict response to immunotherapy in cancer (Bao et al., 2020; Wu et al., 2020), assist diagnosis of cancer through circulating RNA as liquid biopsies, and so on (Mugoni et al., 2022). In order to understand tumorigenesis, development of cancer, and to improve the treatment strategies, it is necessary to study the transcriptome of cancer.

TROY is a type I transmembrane glycoprotein which plays a momentous role in regulating diverse biological activities. It is highly expressed in epithelial tissues and the central nervous system during embryonic development and expressed in the brain and hair follicle in the postnatal organism (Kojima et al., 2000; Hisaoka et al., 2003; Pispa et al., 2008). TROY includes four highly conserved cysteine-rich extracellular regions (ECD) and a unique intracellular region (ICD) containing sequences binding to TNF receptor–associated factor (TRAF) adapter proteins. ICD binds to TRAF adapter proteins for signal transduction and further activates downstream signaling (Eby et al., 2000). Interestingly, TROY was reported to correlate with tumor development in recent studies. Paulino and colleagues affirmed the intracellular region of TROY activated Rac1 by binding to adapter protein Pyk2 to promote the migration and invasion of glioblastoma cells (Paulino et al., 2010). In colorectal tumor, the overexpressed TROY-activated NF-κB signaling contributed to the proliferation of cancer with deregulated β-catenin activity (Schön et al., 2014). The binding of TROY to EGFR enhanced the continuous expression of the EGFR signal by delaying EGFR endocytosis and promoted invasion of glioblastoma cells through TROY-induced activation of NF-κB (Ding et al., 2018). In addition to that, a few research studies demonstrated that TROY promoted cancer therapy resistance. Ding et al. indicated TROY promoted temozolomide resistance through the JAK-STAT3 signal, and another research found TROY expression associated with irradiation resistance in glioblastoma (Loftus et al., 2013; Ding et al., 2020), whereas the role of TROY in lung cancer is still poorly characterized. We demonstrated that TROY was upregulated in both preclinical and clinical samples of EGFR-TKI resistance, and knocking down TROY could partly reduce the resistance. Furthermore, the expression of TROY is associated with poor prognosis in LUAD.

EMT is a fundamental process of the development and metastasis of cancer. The transition from epithelial to mesenchymal states is associated with changes in cell polarity, cell–cell interactions, and basement membrane (Singh et al., 2018). Several research studies have confirmed that EMT contributed to chemotherapy, radiotherapy, and also targeted-therapy resistance in cancer (Fischer et al., 2015; Tulchinsky et al., 2019; Dudas et al., 2020). In this research, we perceived the phenotype transition between parental and gefitinib-resistant cells, which is consistent with previous studies. Following RNA-sequencing, GO analyses of DE mRNAs were conducted to identify the biological function difference: biological processes such as cell adhesion and extracellular matrix organization, cellular components such as membrane and cell junction, and molecular functions such as protein binding showed major difference. GSEA and KEGG analysis demonstrated that DE mRNAs were involved in several EMT-related pathways including focal adhesion, ECM–receptor interaction, and tight junction. Clinical data verified EMT was a vital cause of first-generation EGFR-TKI resistance, too. Although EMT-induced targeted-therapy resistance in NSCLC has been reported in some studies, the underlying mechanisms are still incompletely been understood. Here, we described that the EMT signature was regulated by TROY and attributed to acquired gefitinib-resistance.

In the last few years, the connection between EMT and CSCs has attracted plenty of attention. CSCs are a subset of cancer cells that are capable of self-renewal and multilineage progenitor differentiation which is concerned to be intrinsically resistant to anticancer therapies. In effect, increasing data suggested that cancer therapies often fail to eliminate cancer cells that have entered the CSC state through triggering of the EMT process, consequently permitting CSC-mediated clinical relapse (Shibue and Weinberg, 2017). A recent study in human breast cancer found that CSC plasticity relied on ZEB1, a key regulator of the EMT. In addition, tumor microenvironmental signals such as TGFβ could switch cancer cell states between non-CSC and CSC by controlling the ZEB1 promoter (Chaffer et al., 2013). In this study, we observed that gefitinib-resistant cells highly expressed EMT transcript factor ZEB1 as well. Aldehyde dehydrogenase 1 (ALDH1) was recognized as an essential marker for EGFR-mutation-positive NSCLC CSCs (Codony-Servat et al., 2019); we correspondingly confirmed that ALDH1 expression was significantly elevated in HCC827/GR cells. Cellular quiescence is a nondividing state which is the counterpart to proliferation. The quiescent state CSCs has emerged as an important driver of drug resistance; some lineage-tracing experiments displayed that quiescent CSCs could regenerate the tumor after chemotherapy (Oshimori et al., 2015). In breast cancer, subpopulations of CSCs underwent EMT and not only induced migratory phenotype but also bring about a slow proliferative state which conferred resistance to antiproliferative drugs (Creighton et al., 2009). Kassem et al.’s studies identified that TROY regulates the differentiation fate of human mesenchymal stem cells through canonical Wnt signaling (Qiu et al., 2010). Our data showed that gefitinib-resistant NSCLC cells exhibited EMT features and CSC properties with a slow proliferation rate, which could be reversed by knocking down TROY.

Altogether, these data strongly suggest that TROY plays an essential role in acquired resistance of gefitinib; the expression of TROY indicates shorter overall survival and is an independent prognostic factor in LUAD. Targeting TROY reversed the gefitinib tolerance through the effect of EMT and CSC properties, which established TROY as a promising target in EGFR-TKI resistant NSCLC. However, more studies are needed for the clinical utility of TROY.

## Data Availability

The data presented in the study are deposited in the GEO repository, accession number GSE199627.
